# Synchronous Hodgkin lymphoma and gastric adenocarcinoma: A rare case report and literature review

**DOI:** 10.1097/MD.0000000000009484

**Published:** 2018-01-19

**Authors:** Hongying Wu, Liyan Wei, Lumei Hao, Xuemei Li, Lei Wang, Chenglu Yuan

**Affiliations:** a Qingdao Municipal Hospital (group); b Shandong University Qilu Hospital (Qingdao), Qingdao, Shandong, China.

**Keywords:** adenocarcinoma, gastric, Hodgkin lymphoma, synchronous

## Abstract

Hodgkin lymphoma (HL) is a lymphoproliferative disease arising in the lymphoid tissue, which is characterized by Reed–Sternberg cells. Adenocarcinoma is the most frequent pathological type of stomach cancer. Improved survival in HL patients leads to the development of secondary malignancies. However, synchronous occurrence of these 2 malignancies is extremely rare. Here, we present a 45-year-old male complaining of a lymph node mass in the neck, without any abdominal symptoms, diagnosed as HL and gastric adenocarcinoma with hepatitis B carrier status. We treated the patient with 8 courses of pirarubicin bleomycin, vincristine, and dacarbazine (modified ABVD), and 4 courses of capecitabine therapy concurrently along with oral entecavir, as the patient survived longer than 20 months.

The prognosis of multiple primary malignancies is poor because therapy is difficult, without a standard treatment. The frequency of multiple primary malignancies is increasing in recent years, and second malignancies in patients with cancer should be taken into consideration.

## 1 Introduction

Classical Hodgkin lymphoma (HL) results from malignant transformation of a mature B cell at the germinal center of differentiation and is characterized pathologically by multinucleated Hodgkin and Reed–Sternberg cells embedded in a mixed infiltrate of non-neoplastic cells. Stomach cancer is the most frequent malignant tumor after lung cancer, and accounts for 9% of all cancer cases.^[1]^ Gastric adenocarcinoma represents 85% of all gastric cancers. HL is treated with the intent to cure the disease in all the stages, and long-term survival exceeds 85%.^[2]^ However, secondary cancers are a leading cause of morbidity and mortality among the nearly 175,000 HL survivors. In an international cancer registry study, the relative risk of stomach cancer among HL survivors diagnosed at age 30 years increased 9.5-fold compared with the general population.^[3]^ However, synchronous HL and stomach adenocarcinoma have been reported only in 4 cases.^[4–7]^ In this case, we reported synchronous HL and persistent gastric adenocarcinoma; surgery and sequential chemotherapy may be an effective intervention for such patients.

## 2 Case report

**Figure 1:**
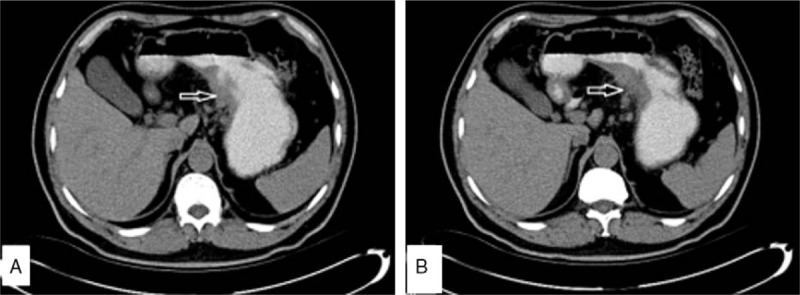
(A,B) CT scan showed wall thickness in the lesser curvature region of the stomach, and limited lymph nodes in the enterocoelia.

**Figure 2:**
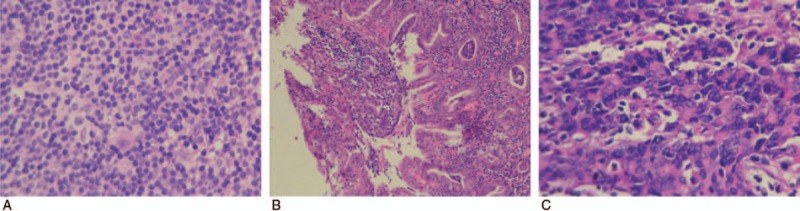
(A) Hodgkin lymphoma with Reed–Sternberg cell variants. HE staining.  × 100. (B) Gastric adenocarcinoma of gastric body HE staining.  × 40. (C) Adenocarcinoma with atypical glands and cells. HE staining.  × 100.

**Figure 3:**
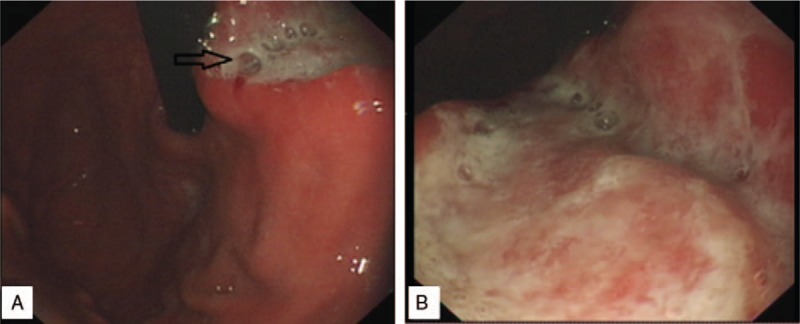
(A,B) A huge ulcer with a malignant appearance in the gastric cardia. Its surface was covered with white mucus.

A 45-year-old man was admitted to the Department of Stomatology with complaints of right neck involving painless lymph node masses that started 20 months ago (March 3, 2015). No history of weight loss or fever, digestive tract symptoms, or peptic ulcer were observed. He was healthy before except for a history of hepatitis B carrier status for about 6 years, without any family tumor history. Physical examination was normal except for a few painless, fixed lymph nodes, measuring 1^∗^2 and 2^∗^2 cm in diameter, in the bilateral neck and axilla, and groin region. Laboratory examination showed lactic dehydrogenase (LDH), hemoglobin, platelets, leukocyte, and other parameters within the normal range. The patient tested positive for hepatitis B virus (HBV) surface antigen. However, no HBV DNA was detected. Bone marrow cellular morphology and flow cytometry results were normal. Abdominal computerized tomography (CT) showed wall thickness in the lesser curvature of the stomach, and limited lymph nodes in the enterocoelia (Fig. 1A, B). No lymph nodes were detected in the mediastinal region. A biopsy of the right neck lymphadenopathy was conducted with open surgery on March 8, 2015. The pathological report was compatible with nodular sclerosis and HL (Fig. 2A). Immunohistochemical staining revealed CD30 (+) CD15 (+) Ki-67 (15%+) CD20 (−) CD3 (−)CD10 (follicle +) CD21 (follicle +) status. Upper gastrointestinal endoscopy showed a huge ulcer with a malignant appearance in the gastric cardia (Fig. 3A, B). Gastric pathology showed gastric adenocarcinoma (Fig. 2B), which tested HP (−) HER-2 (0). Five days later, total gastrectomy and Roux-en-y esophagojejunostomy were performed. Pathological examination showed poorly differentiated adenocarcinomas (Fig. 2C) and a diagnosis of grade II adenocarcinoma (T2N1M0). Accordingly, the patient was diagnosed as stage III AS HL and synchronous stage II gastric adenocarcinoma. After 10 days postsurgery, the patient received a cycle of tegafur-gimeracil-oteracil potassium capsule (s-1) for 1 month, along with continuous oral entecavir therapy to avoid HBV reactivation. The patient was treated with 2 cycles of modified ABVD regimen comprising pirarubicin 40 mg/m^2^ d1, d15; bleomycin 10 mg/m^2^ d1, d15; vincristine 1.4 mg/m^2^ d1, d15, and dacarbazine 375 mg/m^2^ d1,d15, every 28 days. The patient's oncologist and surgeon recommended treatment with another cycle of capecitabine due to side effects associated with oral tegafur-gimeracil-oteracil potassium capsule, especially cutaneous pruritus and pigmentation. Symptoms disappeared with treatment cessation and oral anti-allergic drugs. Therefore, a sequential therapy including 2 cycles of modified ABVD followed by a single cycle of capecitabine was planned. The patient showed adequate compliance and tolerance. The patient was assessed with PET-CT 2 months after completing 8 cycles of modified ABVD and 4 cycles of capecitabine chemotherapy. The results showed shrinkage of lymph nodes on the neck bilaterally, axilla and groin to about 1.9^∗^0.8 cm. Fluorodeoxyglucose (FDG) uptake was slightly increased. The standard uptake value (SUV) max was 1.5. The HBV infection was stable. The patient did not complain of any discomfort and routine follow-up is ongoing as of November 3, 2016.

## 3 Discussion

HL is one of the most common malignancies among adolescents and young adults. Major advances in HL treatment in recent decades have led to dramatic improvements in survival. However, the incidence of secondary cancers is increasing, and leading to higher morbidity and mortality. Secondary primary tumors observed in HL patients have been reported in many studies.^[8–12]^ Chien et al^[13]^ conducted a retrospective study demonstrating an approximately 1.5-fold greater risk of standardized incidence ratios in non-HL (NHL) patients compared with the general population in Taiwan. Second, primary tumors were observed in leukemia, myeloma, and neoplasms of the bone and soft tissue, thyroid, central nervous system, skin, stomach, head and neck, liver and biliary tract, and lungs and mediastinum. Morton et al^[3]^ found that patients with HL who received sub-diaphragmatic radiotherapy showed a dose-dependent increase in the risk of stomach cancer, with marked risks for patients who also underwent chemotherapy containing high-dose procarbazine. The association between synchronous gastric adenocarcinoma and primary gastric lymphoma was related to *Helicobacter pylori* infection.^[14]^ Epstein–Barr virus (EBV) is also related to specific types of lymphoma, nasopharyngeal carcinoma, and gastric carcinoma.^[15]^ A high rate of 25% and 24.13% of HBV DNA was detected among patients with HL and NHL, respectively.^[16]^ However, a few studies demonstrated that HBV was not correlated with gastric carcinoma.^[17]^ Our patient was not treated with radiotherapy or *H. pylori,* and EBV was detected only in hepatitis B carriers. Therefore, we speculated that HL and gastric adenocarcinoma occurred incidentally. The factors contributing to synchronous gastric adenocarcinoma and HL were not clear, underscoring the need for investigation into the relationship between virus infection and tumorigenesis.

Multiple primary cancers are malignant tumor types, manifesting as more than 1 primary tumor diagnosed in the same patient, either simultaneously or sequentially. Synchronous gastric adenocarcinoma and lymphoproliferative disorders have been reported until now. Most of them were diagnosed in combination with gastric NHL.^[18–20]^ Because of the similarity in CT image between gastric carcinoma, mucosa-associated lymphoid tissue (MALT) lymphoma, and HL, histological study of the resected specimens is necessary for differentiation, although synchronous occurrence of HL and gastric adenocarcinoma is extremely rare. To our knowledge, only 4 cases have been previously reported in the literature. Compared with the cases reported previously, our patient was younger and a hepatitis B carrier, with long-term survival. Comez et al^[5]^ presented a 52-year-old male complaining of epigastric pain and fever diagnosed as stage II gastric adenocarcinoma, and IVBS HL similar to our patient. The chemotherapy regimen included a single cycle of FUFA (5-fluorouracil 725 mg days 1–5 and leucovorin 35 mg days 1–5), 3 cycles of modified ECF (epirubucin 50 mg/m^2^, cisplatin 60 mg/m^2^, 5-fluorouracil 1000 mg/m^2^, and leucovorin 15 mg), and 8 cycles of ABVD. Bone metastases were detected at the end of therapy. The patient was followed up for 15 months until the study was published. Our patient who was treated simultaneously with modified ABVD regimen and capecitabine manifested low white blood cell count counts, and was weak to endure intensive chemotherapy. Therefore, a sequence regimen of 2 courses of modified ABVD followed by a single cycle of cepecitabine was designed. Vincristine-induced peripheral neuropathy is also common. The side effect can diminish long-term quality of life. Fortunately, our patient was well tolerated. The results were satisfactory. In the absence of guidelines for the management of such diseases, the treatment modality should be determined according to physician's experience. The characteristics of the patient's illness and performance status need to be closely monitored. We recommend individual therapeutic strategies according to the patient's illness. The therapeutic effects and patient's endurance must be evaluated simultaneously and the case managed using a multidisciplinary approach.

Synchronous multiple primary cancers are difficult to manage because of lack of simultaneous treatment interventions. Treatment choice depends on tumor location and may involve curative surgical resection, radiotherapy, and chemotherapy.^[21]^ In the current case report, the patient underwent a combination of treatments including surgical excision and chemotherapy. The patient was in good health 36 months later. Only 4 of the previously reported cases were followed up, with no mortality reported. Furthermore, due to the limited follow-up data, the relationship between tumor grade, stage, and prognosis remains unclear. Additional cases, therefore, need to be investigated in order to further clarify the key diagnostic and therapeutic characteristics of synchronous neoplasms.

Interestingly, abdominal CT showed few lymph nodes in the enterocoelia. We first hypothesized that it was due to HL, without using endoscopy. Treatment for HL alone results in poor efficacy. In conclusion, newly diagnosed lymph nodes in a cancer patient may represent synchronous or metachronous second primary cancer.
